# Sarcopenia Is an Independent Risk Factor for Severe Diabetic Nephropathy in Type 2 Diabetes: A Long-Term Follow-Up Propensity Score–Matched Diabetes Cohort Study

**DOI:** 10.3390/jcm11112992

**Published:** 2022-05-25

**Authors:** Yen-Min Huang, Wan-Ming Chen, Mingchih Chen, Ben-Chang Shia, Szu-Yuan Wu

**Affiliations:** 1 Division of Hematology and Oncology, Department of Internal Medicine, Hemophilia and Thrombosis Treatment Center, Chang Gung Memorial Hospital, Keelung 204, Taiwan; 8902004@cgmh.org.tw; 2 Division of Hematology and Oncology, Department of Internal Medicine, Lotung Poh-Ai Hospital, Yilan 265, Taiwan; 3 Graduate Institute of Business Administration, College of Management, Fu Jen Catholic University, Taipei 242, Taiwan; daisywanmingchen@gmail.com (W.-M.C.); 081438@mail.fju.edu.tw (M.C.); 025674@mail.fju.edu.tw (B.-C.S.); 4 Artificial Intelligence Development Center, Fu Jen Catholic University, Taipei 242, Taiwan; 5 Department of Food Nutrition and Health Biotechnology, College of Medical and Health Science, Asia University, Taichung 413, Taiwan; 6 Big Data Center, Lo-Hsu Medical Foundation, Lotung Poh-Ai Hospital, Yilan 265, Taiwan; 7 Division of Radiation Oncology, Lo-Hsu Medical Foundation, Lotung Poh-Ai Hospital, Yilan 265, Taiwan; 8 Department of Healthcare Administration, College of Medical and Health Science, Asia University, Taichung 413, Taiwan; 9 Centers for Regional Anesthesia and Pain Medicine, Wan Fang Hospital, Taipei Medical University, Taipei 110, Taiwan

**Keywords:** type 2 diabetes, sarcopenia, nonsarcopenia, severe diabetic nephropathy, prognostic factors

## Abstract

**Simple Summary:**

Diabetic nephropathy is a common cause of chronic kidney disease (CKD) and end-stage kidney disease (ESKD) worldwide and results in tremendous wastage of medical resources. Determining the indicators of diabetic nephropathy, such as sarcopenia, and implementing early interventions to prevent disease progression is crucial. The effect of sarcopenia on the risk of severe diabetic nephropathy in patients with type 2 diabetes (T2DM) remains unclear. This study, to date, has the largest sample size and the longest follow-up period among studies investigating this effect by comparing patients with T2DM with and without sarcopenia. This propensity score–matched, population-based cohort study demonstrated that patients with T2DM and sarcopenia may be at a higher risk of severe diabetic nephropathy than are those without sarcopenia. The patients with T2DM and sarcopenia were at a higher risk of severe diabetic nephropathy than were those without sarcopenia irrespective of age, sex, and diabetes severity. Our results may serve as a valuable reference for relevant government authorities in establishing health policies promoting early detection of sarcopenia and exercise to help patients with T2DM overcome sarcopenia.

**Abstract:**

Background: Diabetic nephropathy is a common cause of chronic kidney disease (CKD) and end-stage kidney disease (ESKD) worldwide and results in tremendous wastage of medical resources. Determining the indicators of diabetic nephropathy, such as sarcopenia, and implementing early interventions to prevent disease progression is crucial. Purpose: The effect of sarcopenia on the risk of severe diabetic nephropathy in patients with type 2 diabetes (T2DM) remains unclear. Patients and Methods: We recruited patients with T2DM and categorized them into two groups, propensity score–matched at a ratio of 1:1, according to whether they had sarcopenia. We subsequently compared the groups’ risk of severe diabetic nephropathy. Results: The matching process yielded a final cohort of 105,166 patients with T2DM (52,583 and 52,583 in the sarcopenia and nonsarcopenia groups, respectively) who were eligible for inclusion in subsequent analyses. According to both the univariate and multivariate Cox regression analyses, the adjusted hazard ratio (aHR) (95% confidence interval) of severe diabetic nephropathy for the sarcopenia diabetes group compared with the control group was 1.10 (1.08–1.13; *p* < 0.001). Conclusion: The patients with T2DM and sarcopenia were at a higher risk of severe diabetic nephropathy than were those without sarcopenia. Our results may serve as a valuable reference for relevant government authorities in establishing health policies to promote early detection of sarcopenia and exercise to help patients with T2DM overcome sarcopenia.

## 1. Introduction

Type 2 diabetes mellitus (T2DM) is the leading cause of chronic kidney disease (CKD) and end-stage kidney disease (ESKD) in the United States and Taiwan [1]. Diabetic nephropathy is a complex and heterogeneous disease with numerous overlapping etiologic pathways, including changes in glomerular hemodynamics [2,3,4], insulin resistance [5,6], oxidative stress and inflammation [7,8], and interstitial fibrosis and tubular atrophy [9]. Similarly, muscle tissue is becoming increasingly recognized as both an endocrine organ and a major contributor to whole-body insulin sensitivity [10,11,12]. Sarcopenia is therefore associated with insulin resistance, T2DM, and metabolic syndrome [11,12]. Acute and chronic inflammatory processes are common in individuals with CKD, especially ESKD. Sarcopenia is also associated with elevated serum inflammatory parameters [13]. Chronic inflammation may play a role in sarcopenia [13].

All forms of vigorous exercise promote improved glucose disposal because muscle glucose uptake during exercise is insulin-independent [14,15]. Regular exercise increases insulin sensitivity [16,17,18] and is therefore crucial to the management and prevention of metabolic syndrome and T2DM [19,20,21,22]. In addition, exercise serves as a partial solution to sarcopenia because it ameliorates mitochondria-derived problems, and resistance exercise enhances muscle mass and function [23]. Therefore, the association between diabetic sarcopenia and diabetic nephropathy must be further explored. If sarcopenia contributes to severe diabetic nephropathy in patients with diabetes, early correction of sarcopenia may prevent disease progression to diabetic nephropathy and dialysis.

Clarifying the association between sarcopenia and diabetic nephropathy in patients with diabetes would be valuable for promoting the early correction of sarcopenia in patients with diabetes and informing relevant government health policies. The findings of the present study may therefore be used to promote early detection and to prevent patients from requiring dialysis in the future, thereby reducing medical resource wastage, and potentially prolonging the life spans of patients with diabetes.

## 2. Patients and Methods

### 2.1. Data Sources and Study Cohort

We used the January 2008–December 2019 data from Taiwan’s National Health Insurance (NHI) Research Database (NHIRD) as the study data. The NHIRD contains all the registration files and details regarding the original claims data of all NHI beneficiaries (approximately 27.38 million individuals). All the NHIRD records—which are encrypted to protect the beneficiaries’ privacy—include detailed outpatient and inpatient claims data, including each patient’s identification number; birth date; sex; disease diagnostic codes according to the International Classification of Diseases, Ninth Revision, Clinical Modification (ICD-9-CM) and International Classification of Diseases, Tenth Revision, Clinical Modification (ICD-10-CM); treatment information; medical costs; dates of hospital admission and discharge; and date of death. All the data sets can be interlinked by using patient identification numbers. Our protocols were reviewed and approved by the Institutional Review Board of the Tzu-Chi Medical Foundation (IRB109-015-B).

### 2.2. Participant Selection

A total of 480,000 patients with T2DM recorded in the NHIRD were initially enrolled in the diabetes cohort. Patients with CKD or ESKD diagnosed before diabetes diagnosis were excluded from the cohorts. We defined sarcopenia according to the previous study from the Taiwan NHIRD [24]. In order to diminish the selection bias of the definition of sarcopenia, we only recorded the sarcopenia from rehabilitation specialists, orthopedics, or family physicians. In Taiwan, the coding of sarcopenia was based on the previous Taiwan study [25]; sarcopenia was defined as the skeletal muscle mass index (SMI) of 2 standard deviations (SDs) or more below the normal sex-specific means for young persons.

### 2.3. Propensity Score Matching and Covariates

After adjustment for confounders, we used a time-dependent Cox proportional hazards model to model the time from the index date to the onset of severe diabetic nephropathy for the patients with diabetes with and without sarcopenia. To minimize the effects of potential confounders when comparing the risk of severe diabetic nephropathy in the sarcopenia and nonsarcopenia groups, the participants were matched according to propensity scores. The matching variables used were age, sex, adapted diabetes complications severity index (aDCSI) score (including the complication categories of retinopathy; nephropathy; neuropathy; cerebrovascular, cardiovascular, peripheral vascular, and metabolic diseases [26]; and income level, urbanization, Charlson comorbidity index (CCI) score, comorbidities (gum and periodontal diseases, peptic ulcers, sleep disorders, conjunctival diseases, proteinuria, hyperuricemia, alcohol-related diseases, obesity, coronary arterial diseases, anemia, asthma, hypertension, and hyperlipidemia), current smoking habits, former smoking habits, and drug use (use of metformin, angiotensin-converting enzyme inhibitors [ACEIs] or angiotensin receptor blockers [ARBs], statins, and insulin). Comorbidities were determined according to the *ICD-9-CM* codes in the records of inpatient visits for the main diagnosis, or if the number of outpatient visits within 1 year was ≥ 2. Continuous variables are presented as means ± standard deviations or medians (first and third quartiles), as appropriate. We matched the participants at a ratio of 1:1 by using the greedy method: propensity score matching (PSM) with a caliper width of 0.2 [27]. Matching is a common technique for selecting controls with identical background covariates (for which the investigator deems necessary to control) to minimize differences among groups of study participants. The primary endpoints were severe diabetes nephropathy. Severe diabetic nephropathy was defined in accordance with National Health Insurance reimbursement regulations [28], which state that treatment with erythropoiesis-stimulating agents can be initiated when patients with chronic kidney disease who do not need dialysis have a serum creatinine concentration greater than 530 μmol/L (approximately equivalent to stage 5 chronic kidney disease) and associated anemia (packed-cell volume < 28%), and maintain a packed-cell volume not exceeding 36% [29]. Thus, patients who were receiving erythropoiesis-stimulating agents covered by health insurance (indicating that serum creatinine concentrations were >530 μmol/L) were considered to have severe nephropathy [30]. In our study, ESKD was defined as the need for a regular course of long-term dialysis or a kidney transplant to maintain life.

### 2.4. Hazard Ratios of Severe Diabetic Nephropathy

We used a Cox model to perform regression on the variables of severe diabetic nephropathy in the sarcopenia and nonsarcopenia groups, and a robust sandwich estimator was used to account for clustering within matched sets [31]. Even if PSM is applied, residual imbalance might still exist in a population [32,33], and a multivariate Cox regression analysis should still be performed. Therefore, we performed a multivariate Cox regression analysis to calculate hazard ratios (HRs) with 95% confidence intervals (CIs) to determine whether the aforementioned factors were independent predictors of severe diabetic nephropathy.

### 2.5. Statistical Analysis

All the analyses were performed using SAS version 9.4 (SAS Institute, Cary, NC, USA). The matching procedure was implemented using PROC PSMATCH in SAS version 9.4 [34]. In a two-tailed Wald test, a *p* value of < 0.05 was considered significant. The cumulative incidence of severe diabetic nephropathy and overall survival (OS) were estimated using the Kaplan–Meier method, and differences between the sarcopenia and nonsarcopenia groups were determined using a stratified log-rank test to compare the groups’ cumulative risk and survival curves (stratified according to matched sets [35]).

## 3. Results

### 3.1. PSM and Study Cohort

The matching process yielded a final cohort of 105,166 patients (52,583 and 52,583 in the sarcopenia and nonsarcopenia groups, respectively) who were eligible for inclusion in subsequent analyses; their characteristics are summarized in Table 1. The age distribution was balanced between the groups (Table 1). Age, sex, aDCSI scores, CCI scores, income levels, comorbidities (gum and periodontal diseases, peptic ulcers, sleep disorders, conjunctival diseases, proteinuria, hyperuricemia, alcohol-related diseases, obesity, coronary arterial diseases, anemia, asthma, hypertension, and hyperlipidemia), current smoking habits, former smoking habits, and drug use (use of metformin, ACEIs or ARBs, and statins) were similar between the groups after head-to-head PSM, and no significant intergroup differences in any of the variables were observed. The crude primary endpoint of severe diabetic nephropathy (advanced-stage diabetic CKD or diabetic ESKD) in the sarcopenia group was significantly different from that in the nonsarcopenia group (*p* < 0.001; Table 1).

### 3.2. Kaplan–Meier Cumulative Incidence of Severe Diabetic Nephropathy and Survival Curves of the Sarcopenia and Nonsarcopenia Groups

**Figure 1:**
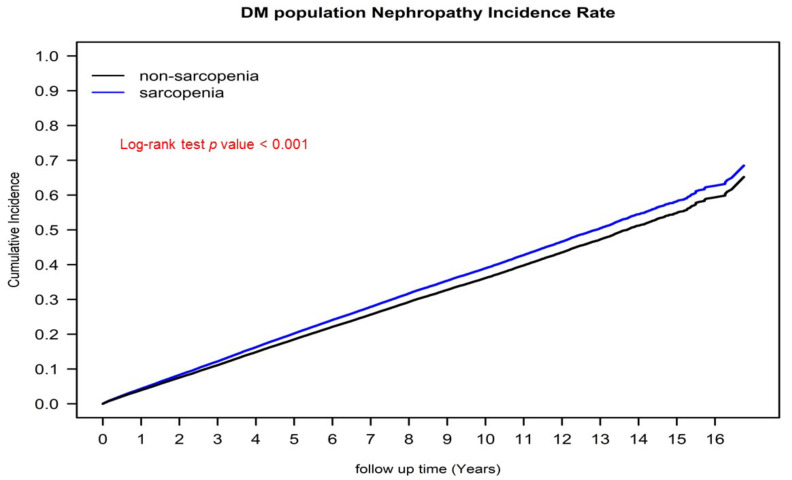
Cumulative incidence of severe diabetic nephropathy in propensity score–matched patients with diabetes with and without sarcopenia.

**Figure 2:**
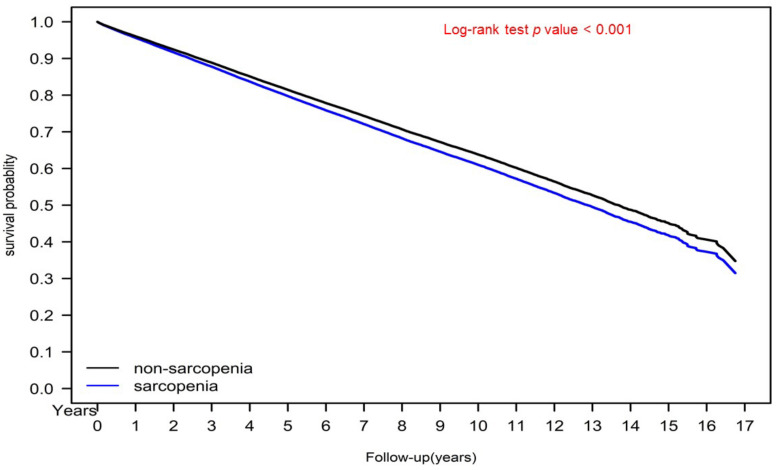
Kaplan–Meier overall survival curves for patients with diabetes with and without sarcopenia.

Figure 1 presents the cumulative incidence of severe diabetic nephropathy for the sarcopenia and nonsarcopenia diabetes groups, as determined using the Kaplan–Meier method. The risk of cumulative severe diabetic nephropathy was significantly higher in the sarcopenia group than in the nonsarcopenia diabetes group. Figure 2 presents the survival curves (in terms of OS) for the sarcopenia and nonsarcopenia diabetes groups, as obtained using the Kaplan–Meier method. The 10-year OS rates for the two groups were 65.31% and 60.97%, respectively (*p* < 0.001).

### 3.3. Prognostic Factors for Severe Diabetic Nephropathy in Multivariate Cox Regression Analysis

**Figure 3:**
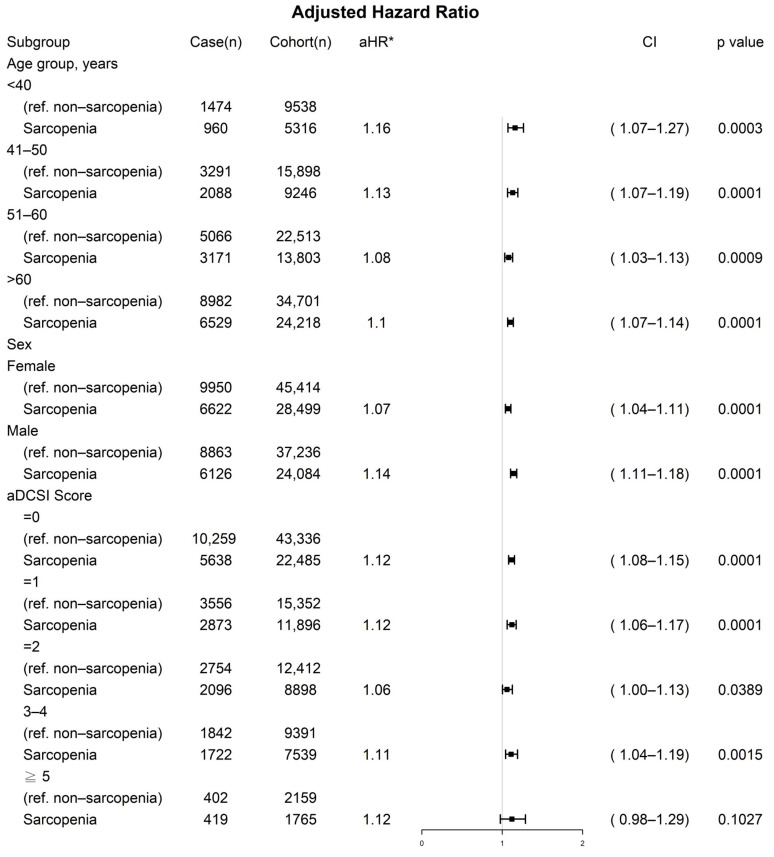
Sensitivity analysis of sex, age, and adapted diabetes complications severity index scores conducted using inverse probability of treatment weighting for severe diabetic nephropathy in patients with diabetes with and without sarcopenia. * Adjusted for all covariates in Table 2.

The results of the multivariate Cox regression analysis indicated that the sarcopenia diabetes group exhibited less favorable prognostic factors for severe diabetic nephropathy than did the nonsarcopenia group (Table 2). No significant differences were observed in the explanatory variables except for age ≥ 40 years, male sex, and aDCSI score ≥ 1. In the multivariate Cox regression analysis, the aHR (95% CI) of severe diabetic nephropathy for the sarcopenia diabetes group compared with the control group was 1.10 (1.08–1.13; *p* < 0.001). The aHRs (95% CIs) of severe diabetic nephropathy for those aged 41–50, 51–60, and > 60 years (compared with those aged ≤ 40 years) were 1.32 (1.26–1.39), 1.55 (1.48–1.63), and 2.14 (2.04–2.24), respectively (Table 2). The aHR (95% CI) of severe diabetic nephropathy for male patients compared with female patients was 1.29 (1.26–1.32). The aHRs (95% CIs) of severe diabetic nephropathy for those with aDCSI scores of 1, 2, 3, 4, and ≥ 5 (compared with those with an aDCSI score of 0) were 1.01 (1.07–1.14), 1.07 (1.03–1.11), 1.09 (1.05–1.15), and 1.36 (1.26–1.47), respectively. The results of the sensitivity analysis of sex, age groups, and aDCSI scores that were determined using the inverse probability of treatment weighting for severe diabetic nephropathy in the patients with diabetes with and without sarcopenia are presented as a forest plot in Figure 3. The aHRs (95% CIs) for the sarcopenia diabetes group (compared with the control group) were significantly associated with a higher incidence of severe diabetic nephropathy, regardless of age group, sex, or aDCSI score.

## 4. Discussion

Sarcopenia is associated with insulin resistance, T2DM, and metabolic syndrome, with the attendant increased risks of cardiovascular disease and stroke [11,12]. Diabetic CKD and ESKD are also associated with insulin resistance [5,6], and in patients with diabetes, glycemic control may deteriorate as kidney function declines [36]. Sarcopenia may serve as an early surrogate marker of the severity of T2DM and diabetic nephropathy, and can be corrected to prevent disease progression to dialysis [23]. However, no data regarding the association between diabetes with or without sarcopenia and severe diabetic nephropathy, including advanced-stage CKD and ESKD, have been previously published. Therefore, we conducted the first head-to-head PSM study to evaluate the risk of severe diabetic nephropathy in patients with diabetes with and without sarcopenia.

The clinical outcomes of diabetic nephropathy are extremely variable [37]. We focused on advanced CKD and ESKD in the present study. The proportion of people with diabetes who have CKD is approximately 25–30% [38]. Whether the natural history and rate of progression of diabetic nephropathy differs according to sarcopenia status remains unclear. In the vast majority of people with T2DM, the onset of diabetic nephropathy occurs after the age of 40 years, and other factors such as age-related senescence of the kidney and hypertension can contribute to kidney function decline to varying degrees [39,40,41]. In addition, T2DM can remain asymptomatic for years, resulting in delayed diagnosis; therefore, the true time of onset of hyperglycemic exposure is usually unknown [42]. If sarcopenia is a risk factor for diabetic nephropathy, early correction of sarcopenia may decrease an individual’s risk of developing severe diabetic nephropathy and prevent disease progression to dialysis, thereby decreasing the financial burden of dialysis on the NHI program.

Among patients with diabetes, risk factors for diabetic nephropathy include older age, African American or American Indian ancestry, Hispanic ethnicity, low socioeconomic status, obesity, smoking, poor glycemic and blood pressure control, and genetic factors [39,40,41,43,44,45,46]. The patients were matched for all the aforementioned confounding factors in the present study to ensure balance between the sarcopenia and nonsarcopenia groups (Table 1). We also accounted for possible risk factors for the severity of diabetes and sarcopenia (Table 1). After PSM, all the covariates were balanced between the groups. We used a robust PSM-based design to ensure homogeneity between the case and control groups in terms of potential confounding variables. Performing a randomized controlled trial (RCT) to evaluate diabetic nephropathy in patients with diabetes with or without sarcopenia is difficult because sarcopenia cannot be treated through tangible intervention [47]. Balancing the confounding factors of severe diabetic nephropathy in patients with diabetes with and without sarcopenia—a main requirement of an RCT design—is impossible [47]. A PSM-based design, such as that used in the current study, can resolve this problem by maintaining balance between the case and control groups in terms of confounding factors in the absence of bias. Moreover, PSM is the recommended standard tool for estimating the effects of covariates in studies where potential bias may be present [27,48]. Although the main advantage of the PSM methodology is the more precise estimation of covariate effects, PSM cannot control for factors not accounted for in the model. Moreover, PSM is predicated on an explicit selection bias for those who can be matched, meaning that individuals who cannot be matched are excluded from the scope of inference. Our study is the first to use a well-designed PSM design mimicking an RCT to investigate the effect of sarcopenia on nephropathy in patients with diabetes.

In our study, sarcopenia, older age, male sex, and an aDCSI ≥ 1 were identified as poor prognostic factors for severe diabetic nephropathy in patients with diabetes (Table 2). Our findings are consistent with those of previous studies [1,49]. Even if PSM is applied, residual imbalance might still exist in a population [32,33]. Increasing age is directly related to the prevalence of diabetic CKD with a decreased glomerular filtration rate, increasing from 8% in the 5th decade to 19% in the 6th decade to 35% in the 7th decade of life [50]. Both CKD in general and diabetic CKD are more common in female individuals [1]. However, compared with female individuals, male individuals are at a significantly higher risk of progression from late-stage CKD to ESKD (HR 1.37, 95% CI 1.17–1.62) [49]. In addition, diabetic retinopathy is a prognostic factor for CKD progression in patients with T2DM [51]. Diabetic retinopathy was reflected in the patients’ aDCSI scores and was adjusted for in our multivariable Cox regression model. In the multivariable Cox regression analysis, the aDCSI score was identified as an independent prognostic factor for severe diabetic nephropathy, and individuals with higher aDCSI scores were at a higher risk of developing diabetic nephropathy (Table 2). This is the first study to identify the aDCSI score as an independent prognostic factor for severe diabetic nephropathy.

In the sensitivity analysis of age, sex, and aDCSI score, sarcopenia remained an independent risk factor of severe diabetic nephropathy for male or female patients of any age with any aDCSI score in the range of 1–5 (Figure 3). Our findings indicate that for patients with diabetes, sarcopenia might be a valuable independent prognostic factor for severe diabetic nephropathy with a similar pathogenesis of insulin resistance [5,6,11,12]. Sarcopenia can be corrected through exercise and improvement of insulin resistance, suggesting that exercise may prevent the progression of severe diabetic nephropathy in patients with diabetes [16,17,18,23]. As indicated in Figure 1, in the present study, the sarcopenia group was at a significantly higher risk of severe diabetic nephropathy than was the nonsarcopenia group. Moreover, the mortality rate in the sarcopenia group was higher than that in the nonsarcopenia group (Figure 2). However, sarcopenia is not reflected in an individual’s aDCSI score. According to our findings, sarcopenia is a risk factor for the progression of diabetes and should therefore be considered in predictive systems for diabetes (like the aDCSI) in the future.

Diabetic nephropathy is a complex and heterogeneous disease with numerous overlapping etiological pathways [2,3,4,5,6,7,8,9]. Hyperglycemia results in the production of advanced glycation end products and reactive oxygen species [2,3,4]. Although hyperglycemia undoubtedly plays a central role [2,3,4], hyperinsulinemia and insulin resistance may also induce pathogenetic mechanisms, possibly accounting for the variation in the histopathology of T2DM [5,6]. Ultimately, alterations in glomerular hemodynamics, inflammation, and fibrosis are primary mediators of kidney tissue damage, although the relative contribution of these mechanisms likely varies among individuals and with the progression of diabetic nephropathy [3]. Insulin resistance in skeletal muscle is also a primary defect in T2DM [52]. Sarcopenia is a risk factor for various frailty-related conditions that occur in older adults [11]. Sarcopenia, independent of obesity, is associated with adverse glucose metabolism, and the association is the strongest in individuals under 60 years of age, suggesting that low muscle mass may be an early predictor of diabetes risk [12]. Given the increasing prevalence of diabetes and the tremendous cost of medical resources associated with severe diabetic nephropathy, the development of interventions to prevent sarcopenia and its metabolic consequences is urgently required. Because insulin resistance and sarcopenia share similar pathogenetic mechanisms [5,6,11,12], exercise undertaken to improve sarcopenia [23] may help attenuate the progression of severe diabetic nephropathy to dialysis. The results of the present study may serve as a valuable reference for relevant government authorities in establishing health policies promoting early detection of sarcopenia and exercise to help patients with T2DM overcome sarcopenia.

This study has the largest sample size and the longest follow-up period of any cohort study investigating the association between severe diabetic nephropathy and sarcopenia in patients with diabetes. In the current study, we used a head-to-head PSM design, mimicking an RCT, to eliminate potential bias. We matched the groups according to severity of diabetes by using the patients’ aDCSI scores and adjusted for the aDCSI score to determine the effect of sarcopenia on severe diabetic nephropathy in the patients with diabetes. Our results revealed that the aHR (95% CI) of severe diabetic nephropathy for the sarcopenia diabetes group compared with the control group was 1.10 (1.08–1.13; *p* < 0.001). The sensitivity analysis indicated that the aHRs (95% CIs) for the sarcopenia diabetes group were significantly associated with mortality regardless of age group, sex, or aDCSI score.

This study has some limitations. First, in this study, participants were only Asians. The relative susceptibility of non-Asian populations to severe diabetic nephropathy remains unclear; therefore, caution should be exercised when extrapolating our results to non-Asian populations. Second, the diagnoses of all comorbidities were based on *ICD-9-CM* or *ICD-10-CM* codes. Nevertheless, the NHIRD reviews charts and interviews patients to verify the accuracy of such diagnoses, and hospitals with outlier charges or practices may be audited and subsequently heavily penalized if malpractice or discrepancies are identified. Accordingly, to obtain crucial information on population specificity and disease occurrence, a large-scale RCT comparing carefully selected patients with sarcopenia diagnosed before diabetic nephropathy and no sarcopenia, although difficult to perform, may be necessary. Third, recent studies report that sodium-glucose cotransporter 2 (SGLT-2) inhibitors may have beneficial effects on the inhibition of kidney progression [53,54]. SGLT-2 inhibitors were approved by the Taiwan Food and Drug Administration in May of 2017 in Taiwan, but were not covered by national health insurance reimbursement regulations. Therefore, there were no SGLT-2 inhibitors use included in the current study. The distributions of the use of SGLT-2 inhibitors were all zero and equal between the case and control groups; thus, there was no selection bias of SGLT-2 inhibitor use between the case and control groups. Fourth, time-varying proteinuria was detected in clinical courses, which may be potentially associated with diabetic kidney disease. Proteinuria (*ICD-9-CM: 791.0*) was recorded in Table 1 and was homogenous between the case and control groups after PSM. Proteinuria as a time-dependent covariate and was adjusted by a time-dependent Cox regression model (Table 2). After adjustment of proteinuria via the time-dependent Cox regression model for the PSM cohorts, the patients with T2DM and sarcopenia were still at a higher risk of severe diabetic nephropathy than were those without sarcopenia. Finally, the NHIRD does not contain information on dietary habits and laboratory data (like glomerular filtration rate or serum creatinine), which may be risk factors for diabetic nephropathy. Despite these limitations, a major strength of this study is its use of a nationwide population-based registry with detailed baseline information. Lifelong follow-up was possible through the linkage of the registry with the national cause of death database. Considering the magnitude and statistical significance of the effects observed in the current study, the study’s limitations are unlikely to have influenced our conclusions.

## 5. Conclusions

The incidence of severe diabetic nephropathy among the patients with T2DM and sarcopenia was higher than that among the patients with T2DM without sarcopenia, irrespective of age, sex, and diabetes severity. Our results may serve as a valuable reference for relevant government authorities in establishing health policies promoting early detection of sarcopenia and exercise to help patients with T2DM overcome sarcopenia.

## Figures and Tables

**Table 1:** Characteristics and outcomes of propensity score–matched patients with diabetes with and without sarcopenia.

	Nonsarcopenia		Sarcopenia		SMD
	*N* = 52,583		*N* = 52,583		
	*N*	%	*N*	%	
Age (mean ± SD)	59.06 ± 15.26		58.96 ± 14.56		0.0070
	59.00 (49.00, 70.00)		59.00 (49.00, 70.00)		
Age (years)	52,583		52,583		0.0000
Age ≤ 40	5316	10.11%	5316	10.11%	
40 ≤ Age ≤ 50	9246	17.58%	9246	17.58%	
50 ≤ Age ≤ 60	13, 803	26.25%	13,803	26.25%	
Age > 60	24,218	46.06%	24,218	46.06%	
Sex	52,583		52,583		0.0000
Female	28,499	54.20%	28,499	54.20%	
Male	24,084	45.80%	24,084	45.80%	
Income Level (NTD)	52,583		52,583		0.0690
Low-Income	668	1.27%	775	1.47%	
≤20,000	34,181	65.00%	32,633	62.06%	
20,001–30,000	10,052	19.12%	11,343	21.57%	
30,001–45,000	5023	9.55%	5224	9.93%	
>45,000	2659	5.06%	2608	4.96%	
Urbanization Level	52,583		52,583		0.1000
Rural	15,494	29.47%	17,947	34.13%	
Urban	37,089	70.53%	34,636	65.87%	
aDCSI Score (mean ± SD)	1.06 ± 1.40		1.24 ± 1.45		0.1210
aDCSI Score	52,583		52,583		0.1640
0	26,681	50.74%	22,485	42.76%	
1	9950	18.92%	11,896	22.62%	
2	8247	15.68%	8898	16.92%	
3–4	6252	11.89%	7539	14.34%	
≥5	1453	2.76%	1765	3.36%	
CCI Score (mean ± SD)	1.02 ± 1.36		1.36 ± 1.98		0.1990
	0.00 (0.00, 2.00)		0.00 (0.00, 2.00)		
CCI Score	52,583		52,583		0.0000
0	27,195	51.72%	27,195	51.72%	
≥ 1	25,388	48.28%	25,388	48.28%	
Comorbidities					
Congestive Heart Failure	3017	5.74%	2651	5.04%	0.031
Dementia	1209	2.30%	1296	2.46%	0.011
Chronic Pulmonary Disease	10,121	19.25%	9710	18.47%	0.020
Rheumatic Disease	1174	2.23%	1478	2.81%	0.037
Liver Disease	10,249	19.49%	10,037	19.09%	0.021
DM With Complications	2201	4.19%	2197	4.18%	0.000
Hemiplegia and Paraplegia	879	1.67%	1225	2.33%	0.047
Renal Disease	60	0.11%	71	0.14%	0.006
AIDS	22	0.04%	17	0.03%	0.002
Cancer	5266	10.01%	7124	13.55%	0.1331
Gum and Periodontal Disease	22,873	43.50%	27,061	51.46%	0.1600
Peptic Ulcer	15,567	29.60%	20,094	38.21%	0.1830
Sleep Disorder	26,231	49.88%	28,981	55.11%	0.1400
Conjunctival Disease	18,788	35.73%	23,459	44.61%	0.1820
Proteinuria	816	1.55%	1053	2.00%	0.0340
Hyperuricemia	2347	4.46%	2785	5.30%	0.0390
Alcohol-Related Disease	2252	4.28%	2674	5.09%	0.038
Obesity	1271	2.42%	1616	3.07%	0.0400
Coronary Arterial Disease	12,107	23.02%	13,825	26.29%	0.0760
Anemia	4468	8.50%	5687	10.82%	0.0790
Asthma	609	1.16%	608	1.16%	0.0000
Hypertension	25,721	48.92%	27,787	52.84%	0.0790
Hyperlipidemia	17,397	33.08%	20,623	39.22%	0.1280
Current Smoking Habits	12,123	23.05%	13,388	25.46%	0.0560
Former Smoking Habits	728	1.38%	1011	1.92%	0.0420
Drug Use					
Metformin	21,117	40.16%	21,724	41.31%	0.0230
Insulin	3410	6.48%	3419	6.50%	0.0003
ACEIs or ARBs	14,048	26.72%	10,612	20.18%	0.1550
Statins	16,468	31.32%	19,091	36.31%	0.1060
					*p* Value
Follow-Up (years; mean ± SD)	7.94 ± 4.18		7.43 ± 4.10		<0.0001
Follow-Up (years; median [IQR, Q1,Q3])	7.46 (2.36, 9.15)		7.79 (1.75, 8.47)		<0.0001
Outcomes					
Severe Diabetic Nephropathy	7169	13.63%	10,723	20.39%	<0.0001
Diabetic Chronic Kidney Disease	4302	8.18%	6434	12.24%	<0.0001
Diabetic End-Stage Kidney Disease	2867	5.45%	4289	8.16%	<0.0001

AIDS: acquired immune deficiency syndrome; CCI: Charlson comorbidity index; SD: standard deviation; SMD: standardized mean difference; NTD: New Taiwan dollars; N: number; ACEI: angiotensin-converting enzyme inhibitor; ARB: angiotensin receptor blocker; IQR: interquartile range.

**Table 2:** Univariable and multivariable Cox proportional regression model of severe diabetic nephropathy in patients with diabetes with and without sarcopenia.

	Crude HR (95% CI)		*p* Value	Adjusted HR * (95% CI)		*p* Value
Sarcopenia (ref. no)						
Yes	1.17	(1.14, 1.2)	<0.0001	1.106	(1.08, 1.13)	<0.0001
Sex (ref. female)						
Male	1.216	(1.19, 1.24)	<0.0001	1.292	(1.26, 1.32)	<0.0001
Age (years; ref. Age ≤ 40)						
40 < Age ≤ 50	1.4	(1.33, 1.47)	<0.0001	1.321	(1.26, 1.39)	<0.0001
50 < Age ≤ 60	1.765	(1.69, 1.85)	<0.0001	1.553	(1.48, 1.63)	<0.0001
Age > 60	2.699	(2.59, 2.82)	<0.0001	2.141	(2.04, 2.24)	<0.0001
Income Levels (NTD; ref. Low-Income)						
≤ 20,000	0.848	(0.77, 1.24)	0.2311	0.896	(0.81, 1.19)	0.2301
20,001–30,000	0.758	(0.68, 1.14)	0.4525	0.822	(0.74, 1.11)	0.5426
30,001–45,000	0.596	(0.54, 1.16)	0.2972	0.76	(0.68, 1.14)	0.3482
>45,000	0.544	(0.49, 1.26)	0.6452	0.704	(0.63, 1.17)	0.3287
Urbanization (ref. rural)						
Urban	0.876	(0.76, 1.29)	0.2352	0.972	(0.95, 1.13)	0.4234
aDCSI Score						
1	1.305	(1.27, 1.34)	<0.0001	1.011	(1.07, 1.14)	0.0012
2	1.572	(1.52, 1.62)	<0.0001	1.073	(1.03, 1.11)	0.0002
3–4	1.821	(1.76, 1.89)	<0.0001	1.095	(1.05, 1.15)	<0.0001
≥ 5	2.539	(2.37, 2.73)	<0.0001	1.36	(1.26, 1.47)	<0.0001
CCI ≥ 1 (ref. 0)	1.313	(0.88, 1.34)	0.1409	1.076	(0.95, 1.1)	0.1247
Comorbidities (ref. no)						
Congestive Heart Failure	1.193	(0.55, 1.63)	0.3405	1.117	(0.68, 1.15)	0.2591
Dementia	1.215	(0.58, 1.25)	0.5016	0.948	(0.91, 1.18)	0.1434
Chronic Pulmonary Disease	1.066	(0.43, 1.51)	0.3942	1.216	(0.88, 1.26)	0.3863
Rheumatic Disease	1.164	(0.61, 1.72)	0.4309	1.16	(0.82, 1.2)	0.2752
Liver Disease	1.314	(0.78, 1.35)	0.3680	1.055	(0.82, 1.09)	0.4233
DM With Complications	0.967	(0.94, 1.19)	0.2181	0.907	(0.88, 1.03)	0.2483
Hemiplegia and Paraplegia	1.293	(0.76, 1.33)	0.4391	1.044	(0.91, 1.07)	0.4236
Renal Disease	1.289	(0.86, 1.33)	0.5925	1.021	(0.99, 1.05)	0.1395
AIDS	1.206	(0.87, 1.24)	0.6320	0.971	(0.94, 1.04)	0.2375
Cancer	1.356	(0.42, 1.23)	0.4051	1.001	(0.97, 1.03)	0.9730
Anemia	1.31	(0.86, 1.37)	0.4827	1.186	(0.94, 1.24)	0.4028
Asthma	1.294	(0.85, 1.46)	0.7921	1.005	(0.89, 1.13)	0.9297
Proteinuria	1.115	(0.58, 1.86)	0.7201	1.194	(0.88, 1.62)	0.5017
Hyperuricemia	1.399	(0.73, 1.47)	0.3294	1.131	(0.87, 1.19)	0.5302
Obesity	0.963	(0.89, 1.04)	0.3465	1.028	(0.95, 1.11)	0.5025
Alcohol-Related Disease	1.222	(0.75, 1.30)	0.4804	1.099	(0.93, 1.16)	0.6553
Coronary Arterial Disease	1.105	(0.57, 1.54)	0.6402	1.028	(0.99, 1.06)	0.0985
Gum and Periodontal Disease	0.973	(0.95, 1.03)	0.1184	0.911	(0.89, 1.03)	0.2116
Peptic Ulcer	1.297	(0.87, 1.33)	0.4781	1.038	(0.91, 1.07)	0.2251
Sleep Disorder	1.313	(0.58, 1.34)	0.5420	1.024	(0.89, 1.05)	0.2674
Conjunctival Disease	1.222	(0.79, 1.25)	0.2508	0.973	(0.95, 1.04)	0.3337
Hypertension	1.181	(0.58, 1.65)	0.2853	1.115	(0.68, 1.15)	0.4492
Hyperlipidemia	1.236	(0.71, 1.27)	0.4903	0.951	(0.92, 1.18)	0.1324
Current Smoking Habits (ref. no)	1.374	(0.94, 1.41)	0.3772	1.01	(0.98, 1.04)	0.4883
Former Smoking Habits (ref. no)	1.282	(0.95, 1.43)	0.7421	1.01	(0.91, 1.13)	0.8532
Drug Use (ref. no)						
Metformin	1.086	(0.75, 1.52)	0.7704	1.021	(0.91, 1.25)	0.4502
ACEIs or ARBs	1.087	(0.94, 1.73)	0.6713	1.069	(0.93, 1.21)	0.6710
Statins	1.036	(0.60, 1.37)	0.5621	1.049	(0.92, 1.08)	0.2235

AIDS: acquired immune deficiency syndrome; CCI: Charlson comorbidity index; NTD: New Taiwan dollars; CI: confidence interval; HR: hazard ratio; ref.: reference group; ACEI: angiotensin-converting enzyme inhibitor; ARB: angiotensin receptor blocker; IQR: interquartile range. * Adjusted for all covariates in Table 2.

## Data Availability

The datasets supporting the study conclusions are included within this manuscript and its additional files.

## Abbreviations

HR: hazard ratio; aHR: adjusted hazard ratio; CI: confidence interval; RCT: randomized controlled trial; PSM: propensity score matching; ICD-9-CM: *International Classification of Diseases, Ninth Revision, Clinical Modification*; ICD-10-CM: *International Classification of Diseases, Tenth Revision, Clinical Modification*; OS: overall survival; CCI: Charlson comorbidity index; ESKD: end-stage kidney disease; IQR: interquartile range; SD: standard deviation; NTD: New Taiwan dollar; N: number; y: years old; aDCSI: adapted diabetes complications severity index; SMD: standardized mean difference; NHI: National Health Insurance; NHIRD: National Health Insurance Research Database; CKD: chronic kidney disease; T2DM: type 2 diabetes; ACEI: angiotensin-converting enzyme inhibitor; ARB: angiotensin receptor blockers.
